# Esophageal Eosinophilia and Eosinophilic Esophagitis in Celiac Children: A Ten Year Prospective Observational Study

**DOI:** 10.3390/nu13113755

**Published:** 2021-10-24

**Authors:** Fernanda Cristofori, Fulvio Salvatore D’Abramo, Vincenzo Rutigliano, Vanessa Nadia Dargenio, Stefania Castellaneta, Domenico Piscitelli, Davide De Benedittis, Flavia Indrio, Lidia Celeste Raguseo, Michele Barone, Ruggiero Francavilla

**Affiliations:** 1 Interdisciplinary Department of Medicine, Pediatric Section “B. Trambusti”, University of Bari “Aldo Moro”, Via Amendola 207, 70126 Bari, Italy; fernandacristofori@gmail.com (F.C.); vincenzo.rutigliano@uniba.it (V.R.); vanessa.dargenio@unifg.it (V.N.D.); scastellaneta@libero.it (S.C.); lidia.raguseo@gmail.com (L.C.R.); 2 Department of Emergency and Organ Transplantation, Section of Gastroenterology, University of Bari “Aldo Moro”, Piazza G. Cesare 11, 70124 Bari, Italy; fulviodabramo@hotmail.it (F.S.D.); michele.barone@uniba.it (M.B.); 3 Department of Emergency and Organ Transplantation, Section of Pathology, University of Bari “Aldo Moro”, Piazza G. Cesare 11, 70124 Bari, Italy; domenico.piscitelli@uniba.it; 4 Department of Information Engineering, University of Pisa, Largo L. Lazzarino 2, 56122 Pisa, Italy; davide.debenedittis@gmail.com; 5 Department of Medical and Surgical Science, University of Foggia, Viale L. Pinto, 71122 Foggia, Italy; flaviaindrio1@gmail.com

**Keywords:** celiac disease, esophageal eosinophilia, eosinophilic esophagitis, eosinophilic gastrointestinal disorders

## Abstract

The association between eosinophilic esophagitis and celiac disease is still controversial and its prevalence is highly variable. We aimed to investigate the prevalence of esophageal eosinophilia and eosinophilic esophagitis in a large group of children with celiac disease, prospectively followed over 11 years. Methods: Prospective observational study performed between 2008 and 2019. Celiac disease diagnosis was based on ESPGHAN criteria. At least four esophageal biopsies were sampled in patients who underwent endoscopy. The presence of at least 15 eosinophils/HPF on esophageal biopsies was considered suggestive of esophageal eosinophilia; at the same time, eosinophilic esophagitis was diagnosed according to the International Consensus Diagnostic Criteria for Eosinophilic Esophagitis. Results: A total of 465 children (M 42% mean age 7.1 years (range: 1–16)) were diagnosed with celiac disease. Three hundred and seventy patients underwent endoscopy, and esophageal biopsies were available in 313. The prevalence of esophageal eosinophilia in children with celiac disease was 1.6% (95% CI: 0.54–2.9%). Only one child was diagnosed as eosinophilic esophagitis; we calculated a prevalence of 0.3% (95% CI: 0.2–0.5%). The odds ratio for an association between eosinophilic esophagitis and celiac disease was at least 6.5 times higher (95% CI: 0.89–47.7%; *p* = 0.06) than in the general population. Conclusion: The finding of an increased number of eosinophils (>15/HPF) in celiac patients does not have a clinical implication or warrant intervention, and therefore we do not recommend routine esophageal biopsies unless clinically indicated.

## 1. Introduction

The coexistence of celiac disease (CD) and eosinophilic esophagitis (EoE) in pediatric patients is still controversial. CD is an autoimmune disorder triggered by the ingestion of gluten in genetically predisposed individuals, leading to a Th1-type immunological response, gut inflammation, and various symptoms. Its prevalence in Western countries is about 1%, and a gluten-free diet (GFD) is the mainstream treatment [1]. EoE is an inflammatory disorder characterized by symptoms of esophageal dysfunction and histological evidence of eosinophil-predominant inflammation in esophageal biopsies. The diagnosis relies on the persistence of symptoms after excluding other causes of esophageal eosinophilia (EsEo) [2].

EoE has been considered rare, but several epidemiological studies have clearly shown an increase in incidence across all ages. In recent years, numerous case reports and observational studies have proposed an association between EoE and CD. Although this correlation was initially documented in children and adults, large population-based trials did not consistently support it [3,4]. Although both diseases are triggered by aberrant immune responses to ingested antigens and are potentially susceptible to dietary removal, differences in the underlying pathophysiological mechanisms [5,6] and the absence of a common genetic background counteract a relationship.

Previously, the diagnosis of EoE was based on histology even in the absence of suggestive symptoms and/or without excluding other causes of esophageal inflammation, leading to a possible overestimation of its incidence [4,5,6,7,8,9,10]. Considering that the presence of eosinophils (>15/HPF) in the general population is expected to be as high as 1.1% [11], a possible hypothesis is that the presence of esophageal eosinophilia on routine biopsies represents an incidental finding [12,13]. The primary outcome was to investigate the prevalence of EoE at CD diagnosis and its clinical implication in children prospectively enrolled in the two referral centers for Pediatric Endoscopy, covering an estimated population of 107,000 children.

## 2. Materials and Methods

We performed an observational study at the Giovanni XXII Children’s Hospital of Bari and San Paolo Hospital of Bari. The Giovanni XXII Children’s Hospital of Bari is the tertiary referral center for Pediatric CD and EoE, covering an estimated population of 107,000 children (National Institute of Statistics—https://www.istat.it accessed on 1 September 2021). Our center and the Pediatric Unit at the San Paolo Hospital of Bari offer pediatric endoscopy for this geographical area.

In 2008 we adopted a diagnostic protocol according to which all children with CD underwent routine esophageal biopsies. All children referred to our units from January 2008 to January 2019 for diagnosis of CD were considered eligible for this prospective study. All patients had a follow-up of at least one year. The following data were recorded in a database: demographic data, personal and family history of atopy, autoimmune disorders and other associated diseases, clinical presentation, laboratory evaluation, endoscopic and histopathological features, and data prospectively collected during patient follow-up (weight, height were measured and clinical information at diagnosis and annually after that). Age- and sex-specific centiles were calculated according to the WHO growth reference.

To investigate CD, serum concentrations of IgA, anti-transglutaminase-IgA (TTG-IgA) and anti-endomysial antibodies (EMA) were tested. A duodenal biopsy was performed in case of persistent antibody positivity. Quantitative detection of TTG-IgA was assessed by an indirect solid-phase enzyme immunoassay test (ORGENTEC Diagnostika; Mainz, Deutschland). The cut-off value was set for values greater than 10 AU. EMA-IgA was determined by indirect immunofluorescence using monkey esophagus sections as the substrate (Euroimmun Italia Diagnostica Medica SRL; Padova, Italia). Dilutions greater than 1:10 were considered positive and titrated. To exclude the presence of selective IgA deficiency (IgA < 0.07 g/L) [14], serum IgA levels were assayed by nephelometry. Class II antigen HLA typing was performed by polymerase chain reaction sequence-specific oligonucleotides using DQ-CD Typing Plus (DiaGene, Palermo, Italy) [15].

Endoscopic esophageal lesions of EoE, including fixed esophageal rings, white exudates or plaque, longitudinal furrows, edema, diffuse esophageal narrowing, were reported [2].

### 2.1. Endoscopy and Histology

Upper gastrointestinal tract endoscopy was performed on a gluten-containing diet by the same physicians (SC; RF). Four biopsy samples from the esophagus (two proximal and two distal irrespective of macroscopic findings) and six from the duodenum/bulb were obtained. The biopsy samples were flattened, orientated and mounted on filter paper, and wholly placed in formalin and processed according to standard procedures. A pathologist (DP), unaware of the clinical and laboratory results, interpreted the samples. CD diagnosis was based on the presence of the coexistence of positivity of TTG-IgA and EMA, the presence of histological lesions according to Marsh classification [16] and normalization of positive serum-specific antibodies on GFD. The International Consensus Diagnostic Criteria for Eosinophilic Esophagitis: Proceedings of the AGREE Conference were used to diagnose EoE; the cut-off for EsEo was set at ≥15/HPF [2]. After 2013, according to the European Society of Gastroenterology, Hepatology and Nutrition (ESPGHAN) guidelines, parents of children and adolescents with signs or symptoms suggestive of CD, TTG-IgA titers with levels > 10 times the upper limit of normal, the positivity of EMA and HLA DQ2 and/or DQ8 were informed about the possibility of avoiding intestinal biopsies. After counselling, decisions were taken on an individual basis. After the confirmation of CD, all patients started a GFD and then followed up at outpatient clinics [1].

The study adhered to the Declaration of Helsinki, and the institutional ethical committee approved the study.

### 2.2. Statistics

Normally distributed grouped data were expressed as the mean (±SD) and compared using paired and unpaired *t*-tests. Non-parametric grouped data are expressed as median (95% CI) and compared with the Mann–Whitney rank-sum test (paired) or Wilcoxon’s signed-rank test (unpaired). Proportionate data were compared with Fisher’s exact test or the χ^2^ test. Differences between groups were analyzed by the use of the two-tailed Student *t*-test for independent samples. A one-way analysis of variance (ANOVA) was used to compare the means of more than two samples; *p* values < 0.05 were regarded as significant. Considering an incidence in the population of CD of 1.4% [17] and EoE of 0.034% [18], we calculated that a sample of 345 children with CD would be required for this study to identify an association between the two conditions with a 90% power, based on an alpha error of 0.05 and a beta error of 0.1. Statistical analysis was performed using SPSS 13.0 (Chicago, IL, USA). We considered that before 2013, children suspected of having CD had upper endoscopy. Still, after this date, the option of not undergoing confirmatory endoscopy was offered and accepted by a significant number of patients/caregivers; we calculated the lower figure of prevalence considering that all patients, for whom the esophageal biopsy was not available, would have been negative.

## 3. Results

**Figure 1:**
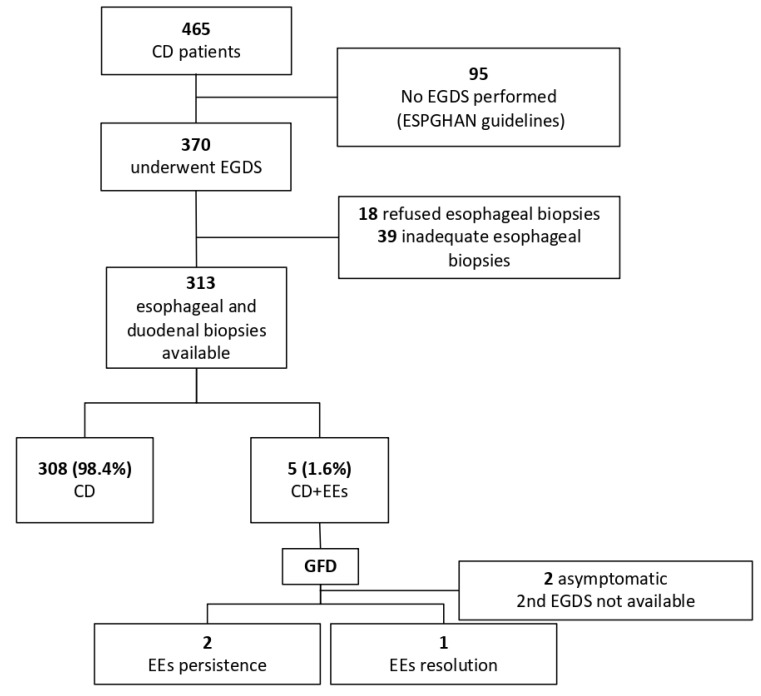
Flow diagram of participants. CD: Celiac disease; EoE: Eosinophilic esophagitis; EsEo: Esophageal eosinophilia; ESPGHAN: European Society of Gastroenterology, Hepatology and Nutrition; GFD: gluten-free diet.

A total of 465 children 195 M (42%); mean age at diagnosis: 7.12 ± 6.2 years (range: 1 to 16) were diagnosed with CD during the study period. Three hundred and seventy patients underwent endoscopy, and esophageal biopsies were available in 313 cases. Endoscopy was not performed in 95 patients according to the ESPGHAN guidelines [1]; no difference was found in the characteristics of biopsied vs. non biopsied individuals. Eighteen patients refused esophageal biopsies, and in 39 cases, the esophageal biopsies were considered inadequate (Figure 1). Patients were followed up for at least one year (mean 1.7 years; range 1–3.4).

### Prevalence of EsEo and EoE at Diagnosis of CD

Two-hundred-ninety esophageal biopsies (93%) showed no eosinophils, 18 (5.4%) mild eosinophilic infiltration (<14 Eos/HPF) and 5 (1.6%) had an EsEo (≥15 Eos/HPF); therefore, the prevalence of EsEo in children with CD was 1.6% (95% CI: 0.54–2.9). If we consider that all patients for whom the esophageal biopsy was not available would have been negative, the lower figure of prevalence would have been 1.07% (95% CI: 0.46–2.5). The characteristics, clinical presentation, laboratory, endoscopic and histological features of children with CD and EsEo are presented in Table 1.

Adherence to a GFD was followed by a progressive resolution of symptoms and normalization of TTG-IgA antibodies. Three celiac patients with EsEo (0.67%; patient 2, 3, 4), who were still symptomatic after six months of strict GFD, underwent a second upper GI endoscopy, and histology showed the persistence of EsEo (>15 Eos/HPF) in two (patient 3, 4). Patients 3 underwent a 24 h pH-impedance monitoring, testing positive for gastro-esophageal reflux. Symptoms improved after a proton pump inhibitor (PPI) treatment, and a repeated endoscopy documented the resolution of EsEo. Only patient four needed topical steroids for the persistence of dysphagia (despite a trial with PPI), with the resolution of symptoms and esophageal eosinophilia. This patient was diagnosed with EoE; therefore, we calculated that the prevalence of EoE in children with CD was 0.3% (1/313; 95% CI: 0.2–0.5%). During the same period, in the same geographical area, we diagnosed EoE in 33 children, leading to a prevalence of EoE in the general population of 0.031% (95% IC: 0.02–0.04). This is similar to the 0.034% (95% CI, 22.3–49.2; I^2^ = 99.7%) recently reported by Navarro P. et al. [12]. The odds ratio for an association between EoE and CD was 9.7 times higher (95% CI: 1.3–71; *p* = 0.03).

We found no difference in auxological (weight and height centiles), nutritional (iron, ferritin, albumin), or biochemical (serum glucose, alanine aminotransferase and hemoglobin levels) parameters and endoscopic appearance between celiac patients with or without EsEo. A higher prevalence of atopy (60% vs. 10.6%; *p* < 0.001) and a lower level of TTG-IgA antibodies (142.5 ± 166 vs. 54.8 ± 26; *p* < 0.02) was found in CD-EsEo as compared to children with CD (Table 2).

## 4. Discussion

We have studied the prevalence of esophageal eosinophils and eosinophilic esophagitis in a representative group of children at CD diagnosis. The presence of EsEo was found in 1.6% of children undergoing esophageal biopsy, while a definite diagnosis of eosinophilic esophagitis was made in one case. Moreover, our study identified that history of atopy and low level of positive TTG-IgA positively correlates with the presence of EsEo; however, these characteristics cannot be a guide in deciding whether to biopsy the esophageal mucosa.

The finding of EsEo did not seem to have a clinical implication or warrant intervention for most patients. The term EoE should be limited to patients with symptoms of esophageal dysfunction and after the exclusion of other causes of esophageal eosinophilia [2].

Several case reports and cohort studies in adults and children have suggested an association between EoE and CD [5,6,7,8,9,10,19,20], although not universally confirmed in extensive population-based studies [3,4]. The figure of prevalence varies widely from 1.2% [21] to 10.7% [7]; however, the retrospective collection of data, absence of power calculation, differences in the definition and management of EoE, the reason for referral, indication for endoscopy, and number and site of esophageal biopsies may explain this variability (Table 3).

Hommeida S. et al., in a retrospective study of medical records of 10,201 children, found 595 with EoE, 546 with CD and 10 with both conditions (1.8%). The authors then calculated an odds ratio of 0.26, arguing against an increased risk of EoE in CD [4]. However, the low number of celiac diagnoses across 17 years and a similar prevalence of EoE and CD in their series is suspicious of a referral bias considering that the prevalence of CD exceeds that of EoE by fifteen times [18]. In contrast, Patton et al. found a prevalence of 6.3% [5]. In their series, 50% of the population had a history of at least one allergic condition; it would be interesting to know if some had been treated with oral allergen immunotherapy that might have increased the risk of EoE [23]. Prinzbach A. et al. performed an unbiased electronic health record-based study and analyzed 433 children with CD matched against 4330 randomly selected controls. The authors found that, besides known comorbidities, among the novel possible associations, EoE was high on the list [24]. Capucilli P. et al. reached a similar conclusion in a 10-year retrospective cross-sectional review of electronic medical records in a single extensive pediatric primary care network (*n* = 456,148), reporting a total of 428 EoE diagnoses and showing a significant association with CD [25].

A limit of the definition of EoE by the updated international consensus diagnostic criteria for eosinophilic esophagitis is what we consider as symptoms of esophageal dysfunction; dysphagia and food impaction are the primary symptoms among adults and adolescents while younger children and infants may present with fewer specific signs, including vomiting, failure to thrive, abdominal and epigastric pain [26]—considering the low median age of participants in our and several other series [4,5,7,8,9,10,12], it might be challenging to apply the proposed definition.

As presented above, the currently available data are controversial and do not answer whether we should routinely biopsy the esophagus of celiac patients. Ahmed et al. found no difference in the percentage of EsEo between 62 children with CD and 91 patients undergoing endoscopy for other indications [12]. The prevalence of EsEo in the pediatric population has been reported in only one study on 28 children showing that eosinophils are rarely detected [27]. The Kalixanda study, performed in a random sample (*n* = 1000) of the adult Swedish population, showed esophageal eosinophils in nearly 5% and the presence of >15 eos/HPF in 1.1% [11]. This is in agreement with our finding that the presence of eosinophils in the esophagus of celiac patients might just be incidental.

The novelty of our data allows us to suggest a practical approach to the pediatric gastroenterologist, that they should proceed to biopsy the esophagus only in the presence of clinical symptoms suggestive of EoE. Indeed, a strategy to biopsy the esophagus in all patients with suspect CD would lead to an overload of costs and difficulty in interpreting the results, with a possible negative impact on the patient.

The second issue to solve is whether or not the association is causal, and a common pathogenesis has not been described yet. A population-based cohort study covering 85% of Utah’s population found an excess risk for multiple autoimmune conditions in subjects with EoE, suggesting a genuine association with CD [28]. No difference in CD-related HLA alleles or polymorphism have been shown in EoE compared to the general population [29]. Immunologically, CD is a TH1-mediated response, while EoE is a TH2-mediated disorder associated with food allergy. TH1 and TH2 immune responses have been considered mutually antagonistic, but one does not exclude the other. Recent molecular studies have indicated that atopic diseases share risk factors that increase inappropriate immune responses, suggesting a more widespread immune dysregulation than a casual association [30,31]. A second theory is that an increased intestinal permeability secondary to CD can promote exposure to various antigens and an up-regulated immune response, which may promote EsEo [32,33,34].

The strengths of our study are the prospective nature of the study, the unselected population of CD patients undergoing esophageal biopsy and the calculation of the statistical power, based on the number of children living in a given area, and served by a given medical center—reinforcing the reliability of our results. Our study has a practical clinical implication and provides a scientific contribution. Firstly, we do not recommend routine esophageal biopsies in celiac children if not clinically indicated. Secondly, we suggest that the finding of EsEo should not be interpreted as EoE, but rather as an incidental finding.

We acknowledge the limitations of our study. A pH study was not routinely performed except in the case of proper clinical indication. A diagnostic proton pump inhibitor trial was not prescribed in all patients [35]; therefore, we cannot be sure that eosinophilia might have, in some cases, been secondary to gastro-esophageal reflux disease. It was not possible to re-biopsy all patients. It is possible that by going gluten-free and eliminating this antigen, some children with EoE may be treated by this dietary elimination. This group would not be captured in the EoE group based on the manuscript’s definition, making the quoted prevalence an underestimate. We are not entirely sure that our centers have identified all children with CD, since some cases might have escaped our direct control because of sanitary migration and the adoption of the new ESPGHAN criteria for CD diagnosis, which might have limited the diagnosis of EoE. The design of the study aimed to evaluate EoE at CD diagnosis, and so does not allow us to know whether a subgroup of these patients might develop EoE over the years. It would be interesting create a register and revaluate patients longitudinally to assess the cumulative incidence of EoE in CD over time.

Finally, although the finding of a higher prevalence of atopy in CD-EsEo is in agreement with the available literature on this topic, we do not have a clear explanation for the finding of low levels of transglutaminase in these patients.

In conclusion, the prevalence of EsEo at CD diagnosis of 1.6% should be considered an incidental finding and should not be considered conclusive for the diagnosis of EoE or limit further dietary restrictions beyond the GFD [36], or lead to unnecessary medical treatment.

## Figures and Tables

**Table 1:** Clinical presentation, endoscopic and histological findings of children with celiac disease and esophageal eosinophilia/eosinophilic esophagitis.

N	Sex	Age (Years)	Symptoms	Atopy	Endoscopic Finding Esophagus Stomach Duodenum	HP	Eosinophil Esophageal Count (n/HPF)	Treatment	Duodenal Histology
1	M	2	AP; Diarrhea	Yes	Normal	Neg	25		Marsh 3a
					Normal			-	
					Scalloping				
2	M	5.6	AP; Diarrhea; Failure to thrive	Yes	Normal	Neg	45		Marsh 3c
					Mild gastritis			-	
					Scalloping				
3	F	3.9	Dyspepsia	No	White exudates	Neg	50		Marsh 3c
					Normal			PPI	
					Scalloping				
4 *	M	5	Dysphagia FI AP	Yes	Esophageal trachealization	Neg	25	swallowed Fluticasone	Marsh 3c
					Normal				
					Scalloping				
5	M	6.4	Anemia	No	Longitudinal furrowing	Neg	40	-	Marsh 3a
					Mild gastritis				
					Scalloping				

AP: Abdominal Pain; FI: Food Impaction; HP: Helicobacter Pylori; HPF: High power field; IgA: Immunoglobulin A; IgE immunoglobulin E; Neg: negative; PPI: proton pump inhibitors; TTG: Tissue transglutaminase. * Patient with CD and eosinophilic esophagitis.

**Table 2:** Auxological, nutritional, biochemical and hematological parameters of children with celiac disease vs. children with celiac disease and esophageal eosinophilia.

	CD (*n*: 308)	CD-EsEo (*n*: 5)	*p* Values
Median Age (range)	7.1 ± 6.3	4.5 ± 1.7	*p* = NS
Gender (F/M)	184/124	1/4	*p* = NS
History of atopy (%)	10.6	60	*p* < 0.001
Weight Centile (mean ± sd)	64.6 ± 31	58.3 ± 24	*p* = NS
Height Centile (mean ± sd)	59.2 ± 27	44.5 ± 28	*p* = NS
Iron (mean ± sd) µg/dL	65.3 ± 34	44.3 ± 33.9	*p* = NS
Ferritin (mean ± sd) ng/mL	29.7 ± 21.7	32 ± 11.5	*p* = NS
Albumin (mean ± sd) g/dL	3.6 ± 0.5	3.2 ± 2.1	*p* = NS
Serum glucose (mean ± sd) mg/dL	85.3 ± 7.1	79.3 ± 6.4	*p* = NS
Hemoglobin (mean ± sd) g/dL	12.2 ± 0.4	11.7 ± 0.5	*p* = NS
Alanine aminotrasferase (mean ± sd) UI/dL	44 ± 13	39 ± 12	*p* = NS
IgE (mean ± sd) g/dL	122 ± 104	51.2 ± 26.2	*p* = NS
Tissue trasglutaminase-IgA (mean ± sd) UI/L	142.5 ± 166	54.8 ± 26	*p* < 0.02

CD: Celiac disease; EsEo: Esophageal eosinophilia; IgE: Immunoglobulin E; NS: Not significant.

**Table 3:** Prevalence of eosinophilic esophagitis in the population with Celiac disease, based on published trials.

First Author, Publication Year	P (*n*)	Study Period	Design	Outcome Indicator	Gender M/F	Age Years Mean (Range)	Response to GFD Clinical ^§^	Response to GFD Histology ^§^
Quaglietta L.—2007 [22]	315	2005–2006	Prospective	1.9% (6 EoE/315 CD)	2⁄4	5.6 (4–10)	6/6 clinical remission	3/3 histological remission
Ooi CY.—2008 [9]	221	2000–2007	Retrospective	3.2% (7 EoE/221 CD)	3/4	5.4 (4.2–10)	1/5 clinical improvement 4/5 clinical remission	1/2 improvement (on GFD + PPI) 1/2 persistent EoE
Leslie C.—2010 [10]	121	1999–2007	Retrospective	4% (10 EoE/250 CD)	6/4	8.5 (2–14)	Not reported	4/4persistent EoE
Abraham JR.—2012 [19]	206	2009–2011	Retrospective	4.4% (9 EoE/206 CD)	4/5	11.3 (8–15)	Not reported	6/98persistent EoE 1/8 persistent EoE ↓eosinophil (GFD + PPI) 1/8 remission (GFD)
Thompson JS.—2012 [6]	297	1981–2012	Retrospective	1.3% (4 EoE/297 CD)	2/2	8 (6–13)	Not reported	Not reported
Stewart MJ.—2013 [21]	245	2004–2008	Retrospective	1.2% (3 EoE)	3/0	13 (11–15)	Not reported	Not reported
Dharmaraj R.—2014 [7]	56	2010–2013	Retrospective	10,7% (6 EoE/56 CD)	5/1	11.6	2/6 improvement (GFD + PPI) 2/6 clinical improvement (GFD+PPI + elimination diet) 1/6 clinical improvement (on GFD, PPI, swallowed fluticasone) 1/6 no improvement (on GFD + PPI + diet)	2/6 remission (on GFD + PPI) 2/6 resolution (GFD + PPI + diet) 1/6 resolution (GFD, PPI, swallowed fluticasone) 1/6 persistent EoE (GFD + PPI + diet)
Ahmed OI.—2015 [12]	220	2007–2012	Retrospective	6.5% (4 EoE/62 CD)	4/0	Not reported	Not reported	Not reported
Hommeida S.—2017 [4]	546	1998–2015	Retrospective	1.86 (10 EoE/V 546 CD); OR: 0.29 (95% CI: 0.15–0.54)	7/3	9 (2–17)	3/10 clinical remission (GFD) 3/10 clinical remission (GFD + steroids + diet) 2/10 clinical remission (GFD + steroids) 1/10 clinical remission (GFD + diet) 1/10 no improvement (not compliant to GFD)	4/4 resolution (on GFD, +swallowed steroid/diet)
Ari A.—2017 [8]	612	2000–2014	Retrospective	5.3 (17 EoE/319 CD)	11/6	5.8 (1–8)	Not reported	3/14 resolution (GFD) 11/14 persistence (GFD)
Patton T.—2019 [5]	350	2008–2013	Retrospective	6.3 (22 EoE/350 CD)	15/7	10.2 (4–17)	Not reported	4/12 resolution (GFD) 8/12 persistence (GFD)

^§^: Number of patients/number of available follow ups. P: Population; CD: Celiac disease; EoE Eosinophilic esophagitis; GFD: Gluten free diet; PPI: Proton pump inhibitor.

## Data Availability

The data presented in this study are available on request from the corresponding author.
